# EASL–EASD–EASO Clinical Practice Guidelines on the management of metabolic dysfunction-associated steatotic liver disease (MASLD): Executive Summary

**DOI:** 10.1007/s00125-024-06196-3

**Published:** 2024-06-13

**Authors:** European Association for the Study of the Liver, European Association for the Study of Diabetes, European Association for the Study of Obesity, Frank Tacke, Paul Horn, Vincent Wai-Sun Wong, Vlad Ratziu, Elisabetta Bugianesi, Sven Francque, Shira Zelber-Sagi, Luca Valenti, Michael Roden, Fritz Schick, Hannele Yki-Järvinen, Amalia Gastaldelli, Roberto Vettor, Gema Frühbeck, Dror Dicker

**Affiliations:** 1 The EASL Building – Home of Hepatology, 7 rue Daubin, CH 1203 Geneva, Switzerland; 2 European Association for the Study of Diabetes e.V., Rheindorfer Weg 3, 40591 Düsseldorf, Germany; 3 EASO Brussels Office, The Library, 10 Square Ambiorix, 1000 Brussels, Belgium

**Keywords:** Diabetes, Glucagon-like peptide, Hepatocellular carcinoma, Liver fibrosis, MASH, MASLD, NAFLD, NASH, Non-invasive tests, Resmetirom

## Abstract

Metabolic dysfunction-associated steatotic liver disease (MASLD), previously termed non-alcoholic fatty liver disease (NAFLD), is defined as steatotic liver disease (SLD) in the presence of one or more cardiometabolic risk factor(s) and the absence of harmful alcohol intake. The spectrum of MASLD includes steatosis, metabolic dysfunction-associated steatohepatitis (MASH, previously NASH), fibrosis, cirrhosis and MASH-related hepatocellular carcinoma (HCC). This joint EASL–EASD–EASO guideline provides an update on definitions, prevention, screening, diagnosis and treatment for MASLD. Case-finding strategies for MASLD with liver fibrosis, using non-invasive tests, should be applied in individuals with cardiometabolic risk factors, abnormal liver enzymes and/or radiological signs of hepatic steatosis, particularly in the presence of type 2 diabetes or obesity with additional metabolic risk factor(s). A stepwise approach using blood-based scores (such as the fibrosis-4 index [FIB-4]) and, sequentially, imaging techniques (such as transient elastography) is suitable to rule-out/in advanced fibrosis, which is predictive of liver-related outcomes. In adults with MASLD, lifestyle modification—including weight loss, dietary changes, physical exercise and discouraging alcohol consumption—as well as optimal management of comorbidities—including use of incretin-based therapies (e.g. semaglutide, tirzepatide) for type 2 diabetes or obesity, if indicated—is advised. Bariatric surgery is also an option in individuals with MASLD and obesity. If locally approved and dependent on the label, adults with non-cirrhotic MASH and significant liver fibrosis (stage ≥2) should be considered for a MASH-targeted treatment with resmetirom, which demonstrated histological effectiveness on steatohepatitis and fibrosis with an acceptable safety and tolerability profile. No MASH-targeted pharmacotherapy can currently be recommended for the cirrhotic stage. Management of MASH-related cirrhosis includes adaptations of metabolic drugs, nutritional counselling, surveillance for portal hypertension and HCC, as well as liver transplantation in decompensated cirrhosis.

**Supplementary Information:**

The online version contains supplementary material available at 10.1007/s00125-024-06196-3.


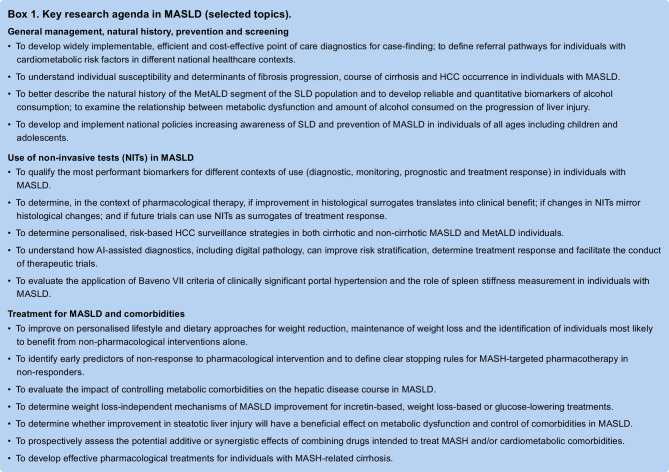


## Introduction

Metabolic dysfunction-associated steatotic liver disease (MASLD) has become the most common chronic liver disease, and its prevalence will likely continue to rise. The presence of MASLD is tightly linked to type 2 diabetes, obesity and other cardiometabolic risk factors. MASLD is associated with an increased risk of cardiovascular events, chronic kidney disease, hepatic and extrahepatic malignancies, and liver-related outcomes, including liver failure and hepatocellular carcinoma (HCC). Therefore, the high socioeconomic burden of MASLD poses a global health challenge that needs to be addressed by medical societies and policymakers [1].

**Fig. 1:**
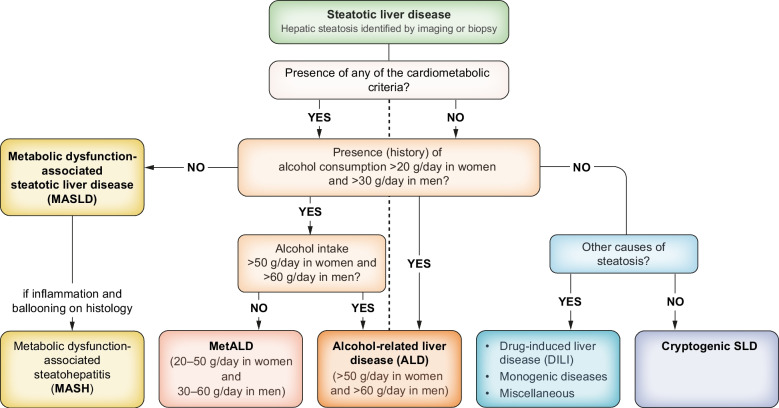
Flow chart for SLD and its sub-categories [2]. SLD, diagnosed histologically or by imaging, has many potential aetiologies. MASLD is defined as the presence of hepatic steatosis in conjunction with (at least) one cardiometabolic risk factor and no other discernible cause. The quantity of alcohol intake, the drinking pattern, and the type of alcohol consumed should be assessed in all individuals with SLD using detailed medical history, psychometric instruments and/or validated biomarkers

MASLD is defined as the presence of excess triglyceride storage in the liver in the presence of at least one cardiometabolic risk factor. The term MASLD comprises different conditions, including isolated liver steatosis (metabolic dysfunction-associated steatotic liver, MASL), metabolic dysfunction-associated steatohepatitis (MASH), as well as fibrosis and cirrhosis. MASH is characterised by histological features of hepatocellular ballooning and lobular inflammation. MASLD replaces the old term non-alcoholic fatty liver disease (NAFLD) and is embedded in the new consensus definition of steatotic liver disease (SLD). Besides MASLD, SLD also includes MASLD with moderate (increased) alcohol intake (MetALD), alcohol-related liver disease (ALD), specific aetiologies of SLD (e.g. drug-induced, monogenic diseases) and cryptogenic SLD (Fig. 1) [2].

The current Clinical Practice Guidelines (CPGs) for the diagnosis, treatment and follow-up of individuals with MASLD have been generated as a joint effort by the European Association for the Study of the Liver (EASL), European Association for the Study of Diabetes (EASD) and European Association for the Study of Obesity (EASO). They update the multi-society NAFLD CPG released in 2016 [3].

Intensified research efforts in recent years have significantly expanded our understanding of the pathophysiology and natural course of the disease. This has culminated in improved diagnostic tools and novel therapeutic options, which is reflected in the expanded scope of the current CPG. The availability of improved treatment options underlines the need to identify at-risk individuals with MASLD early, as we now possess the tools to positively influence the course of the disease, which is expected to prevent relevant clinical events.

These CPGs are targeted at healthcare providers involved in the care of individuals with (or at risk of) MASLD. They provide a framework for the early identification of affected individuals, risk stratification and therapeutic management including non-pharmacological and pharmacological treatment. Furthermore, they provide guidance on the management of end-stage MASLD and MASLD in the setting of advanced liver disease and liver transplantation.

The purpose of this document is to assist physicians, affected and at-risk individuals, healthcare providers and health-policymakers from Europe and worldwide in the decision-making process, by providing evidence-based data, which also takes into consideration the burden of clinical management for the healthcare system. The recommendations are intended to guide clinical practice in circumstances where all possible resources and therapies are available. Thus, users should adapt the recommendations to their local regulations, availability of resources, infrastructure and cost–benefit strategies.

## Preamble

The nomenclature of SLD and definition of MASLD were established in June 2023, following an international, multi-society guided Delphi process [2]. The diagnosis of MASLD requires the presence of at least one cardiometabolic risk factor in an individual with documented steatosis. This has raised concerns as to whether evidence generated under the NAFLD definition would still apply to individuals with MASLD. Several re-examinations from existing cohort studies support that NAFLD-related findings can be fully extrapolated to individuals with MASLD. As an example, analyses of a large tertiary care NAFLD cohort and the population-based Nutrition Examination Survey (NHANESIII) data found a nearly complete overlap between NAFLD and MASLD populations, with 99.8% accordance in the NAFLD cohort, while only 5.3% of individuals with NAFLD in the NHANESIII database did not fulfil the MASLD criteria [4]. In addition, clinical characteristics were almost identical, and non-invasive tests showed equal accuracy and cut-offs for both definitions [4]. Finally, long-term follow-up showed similar mortality rates, with slightly higher mortality in MASLD compared to NAFLD [4]. Therefore, we have transferred the evidence on NAFLD to the MASLD population and use the term MASLD interchangeably. Notably, MetALD represents a distinct entity to which our recommendations and statements generated with the ‘pure’ NAFLD definition may not apply.

## Methods

The levels of evidence (LoE) for all statements and recommendations were developed by applying the Oxford Centre for Evidence-based Medicine system (electronic supplementary material [ESM] Table 1) [5]. The strength of recommendations reflects the quality (grade) of underlying evidence (ESM Table 2). In cases where the committee determined guidance to be necessary despite a lack of available supporting literature, a recommendation was developed based on expert opinion and consensus.

The draft statements and recommendations of the CPG panel were sent to an international 46-member Delphi panel, including clinicians, patient representatives and other stakeholders competent in the field of MASLD, for consensus agreement, where ≥95% agreement was classified as strong consensus and 75–95% were classified as consensus. Neutral votes were not counted when calculating the consensus.

## Definition, prevalence and natural course

*Is the presence of steatotic liver in the general population an important factor in identifying individuals at risk for liver-related outcomes, independent of the presence of other hepatotoxic factors?*

**Recommendations**

- The incidental finding of steatosis should prompt assessment of the potential aetiology of SLD, alongside tests for the presence of advanced fibrosis, as this could determine the risk of liver-related and/or cardiovascular outcomes and appropriate care (**LoE 3, strong recommendation, strong consensus**).
- MASLD, ALD and MetALD are the most common causes of SLD, but other causes such as drug-induced liver disease and monogenic SLD should be considered, depending on the context (**LoE 3, strong recommendation, strong consensus**).
- General population-based screening for SLD is not advised (**LoE 3, strong recommendation, strong consensus**).

**Statement**

- While the presence of steatotic liver in the general population is not independently associated with liver-related outcomes, the stage of liver fibrosis and persistently elevated liver enzymes are associated with liver-related outcomes (**LoE 3, strong consensus**).

A proposal for the simplified diagnostic work-up of a case of SLD is outlined in Fig. 1. Cardiometabolic risk factors and their cut-offs for the definition of MASLD are summarised in ESM Table 3. Other causes of SLD (ESM Table 4) should be considered when all other factors have been excluded (Fig. 1).

*Which risk factors and comorbidities have the greatest impact on the natural history of the hepatic disease including HCC in MASLD?*

**Statements**

- Type 2 diabetes and obesity (particularly abdominal obesity) are the metabolic diseases with the strongest impact on the natural history of MASLD, including progression to MASLD/MASH-related advanced fibrosis, cirrhosis and hepatocellular carcinoma (**LoE 2, strong consensus**).
- Men aged >50 years, postmenopausal women and individuals with multiple cardiometabolic risk factors are at increased risk of progressive fibrosis and the development of cirrhosis and its complications (**LoE 2, strong consensus**).

*Does any alcohol consumption in adults with non-cirrhotic or cirrhotic MASLD have an adverse effect on the natural course of liver disease?*

**Statements**

- Accumulating evidence shows that alcohol consumption and metabolic risk factors have modifying effects on the onset and progression of chronic liver disease, which are independent and can be synergistic (**LoE 2, strong consensus**).
- The presumed beneficial health effects of moderate alcohol consumption are inconsistent across studies, and emerging evidence does not support a protective effect of light to moderate amounts of alcohol, particularly in individuals with cardiometabolic risk factors (**LoE 3, strong consensus**).

**Recommendations**

- The amount, pattern and history of alcohol intake should be documented in all individuals with SLD (**LoE 3, strong recommendation, strong consensus**).
- Alcohol intake may be qualitatively and quantitatively assessed by validated instruments and/or specific biomarkers in individuals with SLD (ESM Table 5) (**LoE 3, open recommendation, strong consensus**).
- Individuals with SLD, particularly those with moderate or high alcohol intake, should be discouraged from consuming alcohol (**LoE 3, strong recommendation, consensus**).
- All alcohol consumption should be stopped completely and permanently in individuals with advanced fibrosis or cirrhosis (**LoE 3, strong recommendation, strong consensus**).

**Prevention**

*In the general population or high-risk groups, can non-pharmacological measures be recommended to prevent the development of MASLD and its adverse complications, including HCC?*

**Recommendation**

- In the general population, non-pharmacological measures should be recommended to prevent the development of MASLD and its complications, including hepatocellular carcinoma, and preventive measures should be reinforced in high-risk groups (**LoE 3, strong recommendation, strong consensus**).

## Screening, case-finding, diagnosis and monitoring

*Should a policy of screening for MASLD at risk of fibrotic disease (or fibrosis progression) in primary care or at the non-hepatology specialist level be implemented in the general population or only in individuals with cardiometabolic risk factors?*

*Which at-risk individuals should undergo case-finding for MASLD at risk of fibrotic disease (or fibrosis progression) in the primary care (or other specialty) setting to reduce hepatic complications of MASLD?*

**Recommendations**

- Healthcare providers may consider case-finding strategies for MASLD with liver fibrosis in individuals with cardiometabolic risk factors (ESM Table 3), abnormal liver enzymes and/or radiological signs of hepatic steatosis (**LoE 3, weak recommendation, consensus**).
- Healthcare providers should look for MASLD with liver fibrosis either in individuals with (1) type 2 diabetes; (2) abdominal obesity and ≥1 additional metabolic risk factor(s) (ESM Table 3); or (3) abnormal liver function tests (**LoE 3, strong recommendation, consensus**).

**Statement**

- Early diagnosis of fibrosis and subsequent appropriate management can potentially prevent progression to cirrhosis and its complications and may justify screening in these populations at risk (**LoE 3, strong consensus**).

*In the adult population with MASLD, are selected non-invasive scores and imaging modalities more useful than liver enzyme testing for the detection of MASLD with fibrosis?*

*In adults with MASLD or at-risk individuals, are clinical care pathways based on the sequential application of non-invasive scores and imaging cost-effective for the identification and management of individuals with MASLD at risk of fibrotic disease (or of fibrosis progression) compared to referral based on physician’s discretion?*

**Recommendations**

**Fig. 2:**
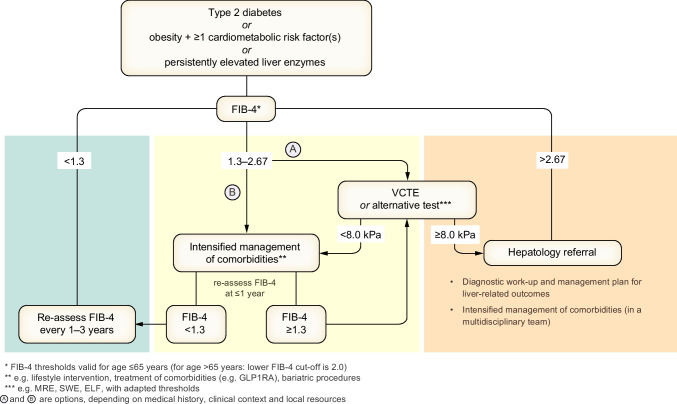
Proposed strategy for non-invasive assessment of the risk for advanced fibrosis and liver-related outcomes in individuals with metabolic risk factors or signs of SLD. Individuals with (1) type 2 diabetes or (2) abdominal obesity and ≥1 additional cardiometabolic risk factor(s) or (3) persistently elevated liver enzymes should undergo a multi-step diagnostic process, as indicated in the figure, to identify individuals with MASLD and advanced fibrosis. The algorithm can also be applied in case of incident finding of steatosis. This strategy is intended to identify individuals at risk of developing liver-related outcomes. MRE, magnetic resonance elastography; SWE, shear wave elastography

- In adults with MASLD, non-invasive scores based on combinations of blood tests or combinations of blood tests with imaging techniques measuring mechanical properties and/or hepatic fat content should be used for the detection of fibrosis since their diagnostic accuracy is higher than standard liver enzyme testing (alanine aminotransferase [ALT] and aspartate aminotransferase [AST]) (**LoE 2, strong recommendation, strong consensus**).
- In adults with MASLD, a multi-step approach is recommended (detailed in Fig. 2): first, an established non-patented blood-based score, such as the fibrosis-4 index (FIB-4), should be used. Thereafter, established imaging techniques, such as liver elastography, are recommended as a second step to further clarify the fibrosis stage if fibrosis is still suspected or in high-risk groups (**LoE 2, strong recommendation, strong consensus**).
- Tests of specific collagen-related blood constituents (e.g*.* enhanced liver fibrosis [ELF]) may serve as an alternative to imaging to identify advanced liver fibrosis (**LoE 2, open recommendation, consensus**).
- Clinical care pathways may be adopted based on the sequential application of non-invasive scores and imaging tests in adults with MASLD or at-risk individuals, recognising that most adults with MASLD are seen in non-hepatology settings (**LoE 2, weak recommendation, strong consensus**).

*In adults with MASLD, should non-invasive scores, circulating biomarkers, liver stiffness measurement and imaging methods replace liver biopsy for the diagnosis of MASH and/or advanced fibrosis?*

**Recommendation**

- Blood biomarker-derived scores and elastography should be used to exclude advanced fibrosis, while elastography is better suited to predict advanced fibrosis (**LoE 2, strong recommendation, consensus**).

**Statements**

- None of these non-invasive methods can assess relevant microscopic features of MASLD such as ballooning or lobular inflammation (**LoE 2, strong consensus**).
- Some blood biomarker-based scores may help to identify individuals with MASH at risk of disease progression (**LoE 3, consensus**).
- Blood biomarker-derived scores and elastography can help in risk stratification for clinical outcomes, as observational studies have identified thresholds related to liver-related outcomes and mortality (**LoE 3, strong consensus**).
- In most cases, liver biopsy is not required for clinical management of individuals with MASLD; however, liver biopsy is still required for the definite diagnosis of steatohepatitis and can help to rule out alternative causes of liver disease (**LoE 1, strong consensus**).

Non-invasively obtained blood-based biomarkers (such as FIB-4 and ELF) and measurements of liver stiffness (vibration-controlled transient elastography [VCTE] or magnetic resonance elastography [MRE]) are suitable for reliably detecting advanced fibrosis with positive and negative predictive values strongly dependent on the chosen cut-off values and the prevalence of fibrosis of different stages in the studied population (Table 1).

**Table 1:**
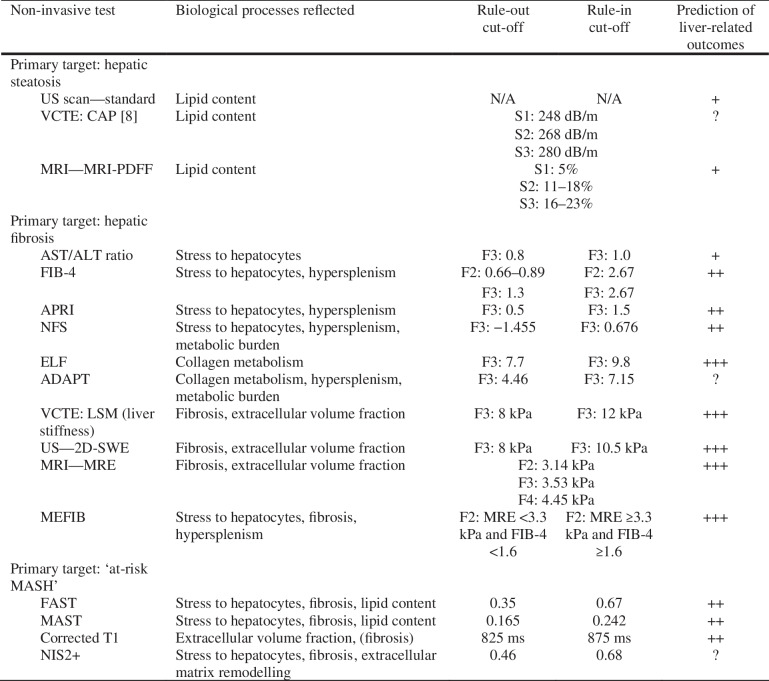
Targets of different non-invasive techniques (selection) and suggested thresholds for ruling out/in certain features of MASLD

The predictive value of the procedure for liver-related outcomes (e.g. cirrhosis complications, HCC, liver-related death) is qualitatively depicted (+ low, ++ moderate, +++ high, ? unknown)

Merged cells represent non-invasive techniques with single cut-offs

ADAPT, age, presence of diabetes, PRO‐C3, and platelet count; APRI, AST to platelet ratio index; AST, aspartate aminotransferase; CAP, controlled attenuation parameter; F1–F4, fibrosis stage (F2: moderate fibrosis, F3: severe fibrosis, F4: cirrhosis); FAST, FibroScan-AST; MAST, MRI-AST; MEFIB, MRE combined with FIB-4; MRE, magnetic resonance elastography; N/A, not applicable; NFS, NAFLD fibrosis score; PDFF, proton density fat fraction; S1–S3, stage of steatosis (S1: mild [<10% hepatocytes], S2: moderate [10%–30% hepatocytes], S3: severe [>30% hepatocytes] steatosis); SWE, shear wave elastography; US, ultrasound

*In adults with MASLD, should non-invasive scores, circulating biomarkers, liver stiffness measurement and imaging techniques be used as a surrogate for liver biopsy to monitor progression of MASH and predict liver-related outcomes?*

**Recommendations**

- In adults with MASLD, sequential assessment with non-invasive tools may assist in ruling out fibrosis progression (**LoE 3, weak recommendation, strong consensus**).
- In adults with MASLD, non-invasive tools (Table 1) can help predict the risk of overall and liver-related events and mortality (**LoE 2, weak recommendation, strong consensus**).

Evidence for the ability of non-invasive tests to predict outcomes in MASLD as well as suggested thresholds for diagnostic purposes are summarised in Table 1.

*In adults with MASLD, does genetic testing (alone or in combination) provide an additional advantage over other non-invasive scores and imaging in predicting risk of liver disease development, severity, progression and liver-related outcomes, or response to specific therapeutic approaches?*

**Recommendations**

- Clinicians in specialised centres may consider assessing the genetic risk profile (e.g. *PNPLA3* p.I148M variant and/or polygenic risk scores) to personalise risk stratification, but this concept should be evaluated in larger prospective studies (**LoE 3, open recommendation, consensus**).
- Genetic risk variants can be evaluated in clinical studies for stratification of disease risk progression and sub-phenotyping of MASLD (**LoE 3, open recommendation, strong consensus**).
- Clinicians can consider referring individuals with a strong family history of severe disease in first degree relatives or early presentation with a severe phenotype, especially in the absence of metabolic triggers (and/or e.g. in individuals with normal body weight), for the evaluation of coexisting, treatable, genetic causes of liver disease by next-generation sequencing approaches (**LoE 4, open recommendation, consensus**).

*Is the assessment of metabolic abnormalities (e.g. insulin sensitivity/resistance) useful for risk stratification or management of adults with MASLD?*

*In adults with MASLD, should diagnostic procedures be performed for associated comorbidities (e.g. cardiovascular diseases, diabetes, dyslipidaemia or obesity)?*

**Recommendations**

- Clinicians should assess associated comorbidities (e.g. type 2 diabetes, dyslipidaemia, hypertension, kidney disease, sleep apnoea, polycystic ovary syndrome) and cardiovascular risk in adults with MASLD (**LoE 2, strong recommendation, strong consensus**).
- At initial diagnosis of MASLD and at regular follow-up intervals, laboratory tests and physical examinations for related comorbidities are recommended (Table 2) (**LoE 2, strong recommendation, strong consensus**).
- Adults with MASLD should be encouraged to participate in extrahepatic cancer screening according to current guidelines, based on their exposure to obesity and type 2 diabetes as risk factors for extrahepatic malignancies (**LoE 3, strong recommendation, strong consensus**).
- Assessment of insulin resistance (e.g. using the homeostasis model assessment of insulin resistance [HOMA-IR] or estimates derived from the oral glucose tolerance test) may be considered to clarify metabolic dysfunction in adults with (suspected) MASLD and without an established diagnosis of type 2 diabetes **(LoE 3, weak recommendation, consensus).**

**Table 2:** Diagnostic procedures to identify relevant comorbidities of MASLD

Comorbidity	Assessment/parameter
Obesity	BMI
	Waist circumference
	Waist to height ratio
	Further investigations^a^:
	Body composition analysis (if available)
	TSH and free thyroxine (if suspicion of hypothyroidism)
Type 2 diabetes or insulin resistance	Fasting plasma glucose
	HbA_1c_
	Oral glucose tolerance test, 2 h post-load glucose
	Fasting plasma insulin and/or C-peptide
	HOMA-IR
	Further investigations^a^:
	Insulin resistance indices from oral glucose tolerance test or mixed meal tests
Dyslipidaemia	Fasting plasma triglycerides
	Fasting plasma total, LDL- and HDL-cholesterol
	Once in a lifetime: measurement of lipoprotein (a)
	Further investigations^a^:
	Non-esterified fatty acids
	Apolipoprotein B
Kidney disease	Creatinine in plasma and urine
	Albumin in serum and urine
	Estimated glomerular filtration rate (eGFR)
Cardiovascular disease	Fasting plasma uric acid
	Serum high-sensitivity C-reactive protein (hsCRP)
	Serum ferritin
	Systolic and diastolic blood pressure
	Further investigations^a^:
	24 h ambulatory blood pressure monitoring
	Echocardiography for heart failure
	Serum NT-ProBNP
	Transferrin saturation
Atherosclerosis	Complete blood count; Platelets
	Elevated lipoprotein (a) is an independent causal risk factor for atherosclerotic cardiovascular disease
	Further investigations^a^:
	Fibrinogen
	Homocysteine
	Von Willebrand factor antigen
	Carotid artery intima media thickness
	EchoDoppler plaque instability
	Coronary artery calcification
Obstructive sleep apnoea	Neck circumference
	Epworth score
	Further investigations^a^:
	Sleep studies
	Overnight pulse oximetry
	Polisomnography
	CPAP trial
PCOS	Sex hormones: LH, FSH, testosterone, SHBG
	Ovarian ultrasound

^a^According to clinical evaluation and a priori probability

CPAP, continuous positive airway pressure; FSH, follicle-stimulating hormone; HDL, high-density lipoprotein; HOMA-IR, homeostatic model assessment of insulin resistance; LDL, low-density lipoprotein; LH, luteinising hormone; NT-ProBNP, N-terminal pro-B-type natriuretic peptide; PCOS, polycystic ovary syndrome; SHBG, sex hormone binding globulin; TSH, thyroid-stimulating hormone

*In adults with non-cirrhotic MASLD or MASH, is surveillance indicated for early detection of HCC?*

**Recommendations**

- In adults with non-cirrhotic MASLD or MASH in the absence of severe fibrosis (i.e. those with fibrosis stage <F3) assessed by non-invasive markers or liver biopsy, surveillance for early detection of hepatocellular carcinoma is currently not recommended (**LoE 3, weak recommendation, consensus**).
- In adults with non-cirrhotic MASLD or MASH in the presence of severe fibrosis (F3) assessed by non-invasive markers or liver biopsy, surveillance may be considered based on an individual risk assessment (**LoE 4, weak recommendation, strong consensus**).

*Should HCC monitoring programmes be implemented in all adults with MASH-related cirrhosis, or should they be implemented based on risk stratification?*

**Recommendations**

- According to current guidelines, hepatocellular carcinoma monitoring programmes should be applied to individuals with MASLD-related cirrhosis (**LoE 3, strong recommendation, strong consensus**).
- Risk stratification can help in optimising strategies for monitoring individuals at higher risk of hepatocellular carcinoma (ESM Tables 6 and 7) (**LoE 4, weak recommendation, strong consensus**).
- As ultrasound-based surveillance has a low sensitivity for detection of hepatocellular carcinoma at an early-stage, particularly in adults with MASLD-related cirrhosis and obesity, alpha-fetoprotein (AFP) measurement can be combined with ultrasound in individuals at high risk (**LoE 3, open recommendation, consensus**).
- Cross-sectional imaging by MRI may be undertaken in selected adults at high risk with persistent poor visualisation at ultrasound, particularly in individuals with dysplastic or regenerative nodules (**LoE 3, open recommendation, strong consensus**).

Algorithms combining demographic and clinical variables with blood-based biomarkers to assess the risk of HCC development are summarised in ESM Table 7. However, none of these calculators have been validated in Phase III/IV studies.

## Treatment of MASLD: general considerations

*In adults with MASLD, which of the following—reduction of steatosis, resolution of MASH, improvement of fibrosis, stabilisation of fibrosis, prevention of progression to cirrhosis—is the most relevant therapeutic target for improving liver-related outcomes?*

**Statements**

- In adults with MASLD and advanced fibrosis or cirrhosis, regression of fibrosis has been associated with a reduced risk of liver-related outcomes (**LoE 2, strong consensus**).
- Improvement in disease activity and resolution of steatohepatitis have been associated with regression of fibrosis (**LoE 2, strong consensus**).
- Reduction of steatosis has been associated with histological improvements (particularly necro-inflammation) in some pharmacological intervention studies (**LoE 2, strong consensus**).
- Since improved mortality has not been demonstrated for any of these treatment-induced histological changes, further long-term follow-up studies are needed to demonstrate that halting disease progression and/or reduction of steatosis, resolution of steatohepatitis or regression of fibrosis translate into a reduced risk of clinical outcomes (**LoE 3, strong consensus**).

*In adults with MASLD, should non-invasive scores, serum markers, liver stiffness measurements and imaging be used as a substitute for liver biopsy for monitoring therapeutic responses?*

**Statements**

- Non-invasive tests have been linked with histologically assessed treatment response, but the most appropriate non-invasive test may depend on the type of intervention and patient-related factors (**LoE 2, strong consensus**).
- Longitudinal changes in non-invasive test results have been correlated with changes in the risk of adverse outcomes on a cohort or population level (**LoE 3, consensus**).
- In the setting of randomised controlled trials, and depending on the mode of intervention, changes of non-invasive markers (e.g. MRI-estimated proton density fat fraction [MRI-PDFF] relative reduction by ≥30%, ALT reduction by ≥17 U/l) have been associated with resolution of steatohepatitis (**LoE 2, strong consensus**).
- Liver biopsy is not suited for monitoring disease evolution or response to therapy in routine clinical practice due to its invasiveness and procedure-related limitations (**LoE 5, strong consensus**).

**Recommendations**

- At the individual level, non-invasive tests may be repeatedly used to assess fibrosis progression in a tailored fashion but may provide limited information about treatment response (**LoE 5, weak recommendation, strong consensus**).
- In individual cases and in clinical trials, liver biopsy can be used to monitor disease progression or response to treatment (**LoE 1, open recommendation, strong consensus**).

*In adults with MASLD, can the management of liver disease and extrahepatic comorbidities within multidisciplinary teams involving hepatologists and other specialists improve clinical outcomes?*

**Recommendation**

- Given the multidirectional connections between MASLD and cardiometabolic comorbidities, a multidisciplinary approach is recommended to ensure all components are appropriately targeted to improve both liver-related and extrahepatic outcomes (**LoE 3, strong recommendation, strong consensus**).

## Treatment of MASLD: non-pharmacological therapy

*In adults with MASLD, what is the efficacy of dietary and behavioural therapy-induced weight loss on histologically/non-invasively assessed liver damage/fibrosis and liver-related outcomes compared with no intervention?*

**Recommendations**

- In adults with MASLD, dietary and behavioural therapy-induced weight loss should be recommended to improve liver injury, as assessed histologically or non-invasively (**LoE 1, strong recommendation, strong consensus**).
- In adults with MASLD and overweight, dietary and behavioural therapy-induced weight loss should aim at a sustained reduction of ≥5% to reduce liver fat, 7–10% to improve liver inflammation and ≥10% to improve fibrosis (**LoE 2, strong recommendation, strong consensus**).

**Statement**

- Further follow-up studies are needed to determine the long-term effectiveness of dietary and behavioural therapy-induced weight loss (including its magnitude) on clinical liver-related outcomes and liver-related mortality (**LoE 3, strong consensus**).

**Fig. 3:**
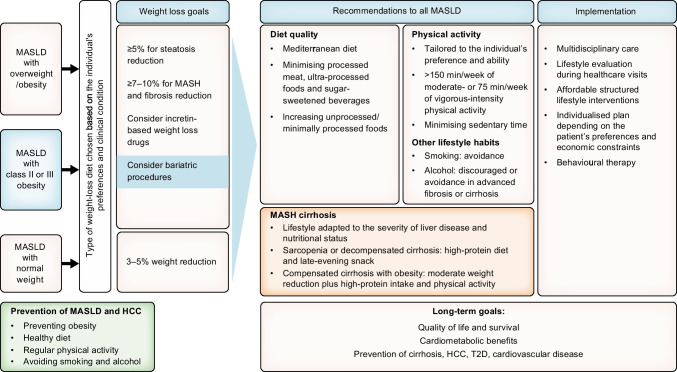
Lifestyle management algorithm for MASLD. Behavioural therapy includes: self-monitoring, clinicians providing affected individuals with self-efficacy and motivation, setting realistic negotiable goals and overcoming barriers. Examples of unprocessed/minimally processed foods: vegetables, fruits (not juice), low-fat dairy, nuts, olive oil, legumes, unprocessed fish and poultry. Overweight/obesity: Overweight: BMI of 25–29.9 kg/m^2^ (non-Asian) or 23–24.9 (Asian), Obesity: ≥30 kg/m^2^ (non-Asian) ≥25 kg/m^2^ (Asian). Class II obesity: BMI ≥35 kg/m^2^ (non-Asian) or BMI ≥30 kg/m^2^ (Asian). Normal weight: BMI<25 kg/m^2^ (non-Asian) or <23 kg/m^2^ (Asian). T2D, type 2 diabetes

Figure 3 gives an overview of the lifestyle management for individuals with MASLD.

*In adults with MASLD, is changing diet quality effective in reducing histologically/non-invasively assessed liver damage/fibrosis and liver-related outcomes compared with no intervention?*

**Recommendation**

- In adults with MASLD, improving diet quality (similar to the Mediterranean dietary pattern), limiting the consumption of ultra-processed food (rich in sugars and saturated fat) and avoiding sugar-sweetened beverages should be recommended to improve histologically or non-invasively assessed liver injury (**LoE 2, strong recommendation, strong consensus**).

**Statement**

- There is little evidence that improving diet quality beneficially impacts clinical liver-related outcomes (**LoE 3, consensus**).

*In adults with MASLD, are physical activity and exercise effective at reducing histologically/non-invasively assessed liver damage/fibrosis and liver-related outcomes compared with no intervention?*

**Recommendation**

- In adults with MASLD, physical activity and exercise should be recommended to reduce steatosis, tailored to the individual’s preference and ability (preferably >150 min/week of moderate- or 75 min/week of vigorous-intensity physical activity) (**LoE 1, strong recommendation, strong consensus**).

**Statement**

- In comparison to the well-documented cardiometabolic benefits, there is less robust evidence for benefits of physical activity and exercise on histological outcomes, non-invasively assessed liver damage/fibrosis and liver-related clinical outcomes (**LoE 5, strong consensus**).

*In adults with MASLD who are normal weight, are diet and exercise interventions effective in reducing histologically/non-invasively assessed liver damage/fibrosis and liver-related outcomes in comparison with no intervention?*

**Recommendation**

- In normal-weight adults with MASLD, diet and exercise interventions should be recommended to reduce liver fat (**LoE 3, strong recommendation, strong consensus**).

**Statement**

- In normal-weight adults with MASLD there is currently no evidence regarding the beneficial effect of diet and/or exercise on liver histology, fibrosis and liver-related clinical outcomes (**LoE 5, consensus**).

*In adults with MASLD, are nutraceuticals (food supplements, herbal products, gut microbiota-modifying agents) effective to reduce histologically/non-invasively assessed liver damage/fibrosis and liver-related outcomes compared with no intervention?*

**Recommendation**

- In adults with MASLD, nutraceuticals cannot be recommended since there is insufficient evidence of their effectiveness in reducing histologically/non-invasively assessed liver damage/fibrosis and liver-related outcomes in MASLD, nor of their safety (**LoE 2, open recommendation, strong consensus**).

**Statement**

- In adults with MASLD, coffee consumption has been associated with improvements in liver damage and reduced liver-related clinical outcomes in observational studies (**LoE 4, strong consensus**).

## Treatment of MASLD: pharmacological therapy

*In adults with MASH, is there sufficient evidence to recommend prescription of existing non-glucose-lowering drugs to reduce histologically/non-invasively assessed liver damage/fibrosis and liver-related outcomes compared to no pharmacological intervention?*

**Recommendations**

- If approved locally and dependent on the label, adults with non-cirrhotic MASH with significant liver fibrosis (stage ≥2) should be considered for treatment with resmetirom as a MASH-targeted therapy, as this treatment demonstrated histological efficacy on steatohepatitis and fibrosis in a large Phase III registrational trial with an acceptable safety and tolerability profile (**LoE 2, strong recommendation, consensus**).
- Treatment with resmetirom, if approved locally, may be considered for individuals with MASLD who are non-cirrhotic and with documentation of either: (1) advanced fibrosis; (2) at-risk steatohepatitis with significant fibrosis (by liver biopsy, when available, or by non-invasive panels validated for that purpose); or (3) risk of adverse liver-related outcomes (e.g*.* by elastography- or biomarker-defined thresholds) (**LoE 3, open recommendation, consensus**).
- No MASH-targeted pharmacotherapy can currently be recommended for adults with MASH at the cirrhotic stage (**LoE 5, weak recommendation, strong consensus**).
- Given the lack of robust demonstration of histological efficacy on steatohepatitis and liver fibrosis derived from large Phase III trials and potential long-term risks, vitamin E cannot be recommended as a MASH-targeted therapy (**LoE 2, weak recommendation, strong consensus**).

**Statement**

- For individuals with MASLD undergoing therapy with resmetirom, data on sustainability of histological benefits, individual prediction of response, liver-related outcomes and long-term safety are not currently available (**LoE 5, strong consensus**).

*In adults with MASH, is there sufficient evidence to recommend prescription of existing glucose-lowering drugs to reduce histologically/non-invasively assessed liver damage/fibrosis and liver-related outcomes compared to no pharmacological intervention?*

**Recommendations**

- In the absence of a formal demonstration of histological improvement in large, well conducted, Phase III trials, glucagon-like peptide 1 receptor agonists (GLP1RA) cannot currently be recommended as MASH-targeted therapies (**LoE 5, strong recommendation, strong consensus**).
- GLP1RAs are safe to use in MASH (including compensated cirrhosis) and should be used for their respective indications, namely type 2 diabetes and obesity, as their use improves cardiometabolic outcomes (**LoE 2, strong recommendation, strong consensus**).
- Where available, pioglitazone is safe to use in adults with non-cirrhotic MASH but given the lack of robust demonstration of histological efficacy on steatohepatitis and liver fibrosis in large Phase III trials, pioglitazone cannot be recommended as a MASH-targeted therapy (**LoE 2, weak recommendation, consensus**).
- There is insufficient evidence to recommend the use of sodium–glucose cotransporter-2 (SGLT2) inhibitors or metformin as MASH-targeted therapies; however, they are safe to use in MASLD and should be used for their respective indications, namely type 2 diabetes, heart failure and chronic kidney disease (**LoE 3, strong recommendation, strong consensus**).

**Statements**

- In case of substantial weight loss induced by GLP1RAs, a hepatic histological benefit could be expected, although this has not been extensively documented so far (**LoE 2, strong consensus**).
- There is insufficient evidence to support using any other glucose-lowering drug class as MASH-targeted therapies (**LoE 5, strong consensus**).

**Fig. 4:**
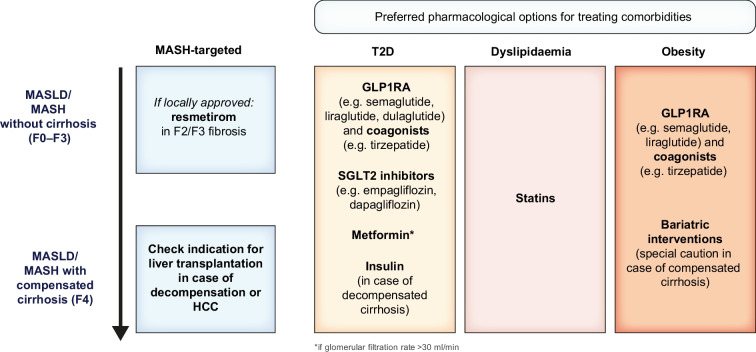
Treatment recommendations beyond lifestyle modification in MASLD/MASH. The recommended choice of pharmacological treatment options in individuals with MASLD/MASH is dependent on comorbidities and stage of disease. T2D, type 2 diabetes

Figure 4 summarises the recommended choice of pharmacological treatment options in individuals with MASH, depending on comorbidities and stage of disease.

*In adults with MASH, is there sufficient evidence to recommend prescription of existing weight-loss agents to reduce histologically/non-invasively assessed liver damage/fibrosis and liver-related outcomes compared to no pharmacological intervention?*

**Recommendation**

- Non-incretin-based weight-loss agents are not recommended as MASH-targeted therapies (**LoE 5, strong recommendation, strong consensus**).

## Treatment of MASLD: surgical and endoscopic therapy

*In adults with MASLD and obesity, are bariatric/metabolic surgery procedures or endoscopic weight-loss interventions effective to reduce histologically/non-invasively assessed liver damage and liver-related outcomes compared with no intervention?*

**Recommendations**

- In adults with non-cirrhotic MASLD who have an approved indication, bariatric surgery should be considered, because it can induce long-term beneficial effects on the liver and is associated with remission of type 2 diabetes and improvement of cardiometabolic risk factors (**LoE 3, strong recommendation, strong consensus**).
- In adults with MASLD-related compensated advanced chronic liver disease/compensated cirrhosis who have an approved indication, bariatric surgery can be considered but careful evaluation (indication, type of surgery, presence of clinically significant portal hypertension) by a multidisciplinary team with experience in bariatric surgery in this particular population is required (**LoE 4, weak recommendation, strong consensus**).
- Metabolic/bariatric endoscopic procedures require further validation as MASH-targeted therapy and cannot currently be recommended (**LoE 4, weak recommendation, strong consensus**).

## End-stage liver disease and liver transplantation

*In adults with MASH-related cirrhosis, should dietary and lifestyle recommendations be adapted to the severity of liver disease, nutritional status and sarcopenia?*

**Recommendations**

- In adults with MASH cirrhosis, it is recommended that dietary and lifestyle recommendations be adapted to the severity of liver disease, nutritional status and the presence of sarcopenia/sarcopenic obesity (**LoE 2, strong recommendation, strong consensus**).
- In adults with sarcopenia, sarcopenic obesity or decompensated cirrhosis, it is recommended that a high-protein diet is provided, as well as a late-evening snack (**LoE 2, strong recommendation, consensus**).
- Moderate weight reduction can be suggested in adults with compensated cirrhosis and obesity, with an emphasis on high protein intake and physical activity to maintain muscle mass and reduce the risk of sarcopenia (**LoE 3, weak recommendation, strong consensus**).

The approach of the majority of nutritional interventions in cirrhosis is to supply at least 35 kcal/kg of body weight/day, with a daily recommended protein intake of 1.2–1.5 g/kg of body weight/day, and recommended strategies are summarised in ESM Table 8.

*In adults with MASH-related cirrhosis, how should pharmacologic interventions for diabetes and lipid control or cardiovascular prevention be adapted to the severity of the liver condition?*

**Recommendations**

- Metformin can be used in adults with compensated cirrhosis and preserved renal function but should not be used in adults with decompensated cirrhosis, especially when there is concomitant renal impairment, because of the risk of lactic acidosis (**LoE 3, strong recommendation, strong consensus**).
- Sulfonylureas should be avoided in adults with hepatic decompensation because of the risk of hypoglycaemia (**LoE 4, weak recommendation, strong consensus**).
- GLP-1 receptor agonists can be used in adults with Child–Pugh class A cirrhosis, according to its indication (**LoE 2, weak recommendation, strong consensus**).
- SGLT2 inhibitors can be used in adults with Child–Pugh class A and B cirrhosis (**LoE 4, weak recommendation, consensus**).
- Statins can be used in adults with chronic liver disease, including those with compensated cirrhosis; they should be used in adults according to cardiovascular risk guidelines to reduce cardiovascular events (**LoE 1, strong recommendation, strong consensus**).

*In adults with MASLD, can non-invasive scores, serum markers, liver stiffness measurements and/or imaging replace hepatic venous pressure gradient (HVPG) and endoscopy in identifying individuals with clinically significant portal hypertension and varices requiring treatment, respectively?*

**Recommendations**

- Liver stiffness measurement (LSM) by vibration-controlled transient elastography (VCTE) ≤15 kPa plus platelet count ≥150×10^9^/l may be used to rule out clinically significant portal hypertension (CSPH) in adults with MASLD (**LoE 3, weak recommendation, strong consensus**).
- If CSPH is present, non-selective beta-blockers may be started unless contraindicated (**LoE 3, weak recommendation, strong consensus**).
- In adults with compensated advanced chronic liver disease but LSM ≥20 kPa and/or platelet count <150×10^9^/l, an upper gastrointestinal endoscopy should be performed to screen for varices unless they already fulfil the criteria to initiate non-selective beta-blockers (**LoE 3, strong recommendation, strong consensus**).

**Statement**

- The threshold of LSM ≥25 kPa to rule in CSPH is only applicable to non-obese (BMI <30 kg/m^2^) adults with MASLD; while obesity can confound LSM, current evidence is insufficient to suggest the optimal non-invasive test to rule in CSPH in adults with MASLD and obesity **(LoE 3, strong consensus**).

*In adults with MASLD who are candidates for liver transplantation, should the evaluation of (cardiometabolic) comorbidities in the pre- and post-transplant phase be different from that of individuals with liver disease of other aetiologies?*

**Statement**

- Adults with MASLD are at increased risk of major cardiovascular events in the pre-, peri- and post-transplant phase (**LoE 2, strong consensus**).

**Recommendations**

**Fig. 5:**
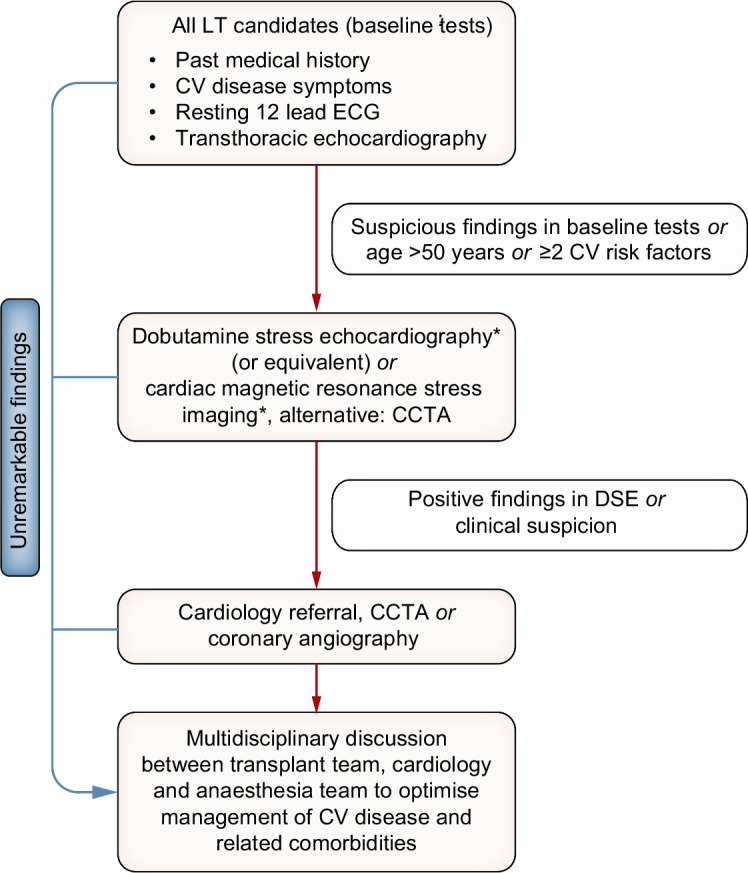
Cardiovascular work-up algorithm in the evaluation of individuals with MASLD before liver transplantation. Adults with MASLD who are candidates for liver transplantation should be evaluated by a multidisciplinary team using a stepwise and risk-adjusted cardiac work-up algorithm to mitigate the risk of major cardiovascular events in the pre-, peri- and post-transplant phase (modified from [6, 7]). CCTA, coronary computed tomography angiography; CV, cardiovascular; DSE, dobutamine stress echocardiography; ECG, electrocardiogram; LT, liver transplantation; TTE, transthoracic echocardiography. *Indicates suboptimal sensitivity in high-risk populations

- Adults with MASLD who are candidates for liver transplantation should be evaluated by a multidisciplinary team for cardiovascular and metabolic comorbidities to mitigate the risk of major cardiovascular events in the pre-, peri- and post-transplant phase (**LoE 3, strong recommendation, strong consensus**).
- A comprehensive screening for comorbidities in adults with MASLD before liver transplantation (ESM Table 9), including a stepwise and risk-adjusted cardiac work-up algorithm (Fig. 5), may help to optimise management of adults with MASLD before, during and after liver transplantation (**LoE 5, weak recommendation, strong consensus**).

*In potential liver transplant recipients with MASH and severe obesity, do pharmacologic treatments, endoscopic interventions and bariatric surgery for weight loss improve outcomes before and after transplantation?*

**Recommendations**

- Adults with obesity and end-stage MASLD listed for liver transplantation should undergo therapeutic interventions aimed at weight reduction without worsening sarcopenia as this will improve peri-operative outcomes (**LoE 3, strong recommendation, strong consensus**).
- Implementation of dietary modification and supervised physical exercise should be the first line management approach with the objective of reducing BMI <40 kg/m^2^ and ideally <35 kg/m^2^ (**LoE 1, strong recommendation, strong consensus**).
- In adults with end-stage MASLD listed for liver transplantation, pharmacological weight-loss strategies may be considered after careful risk–benefit assessment (e.g. presence of sarcopenia, liver function impairment) (**LoE 4, weak recommendation, consensus**).
- In adults with compensated cirrhosis and without clinically significant portal hypertension, sleeve gastrectomy prior to liver transplantation may be considered as an alternative option to dietary or pharmacological weight loss (**LoE 3, open recommendation, strong consensus**).
- In case of decompensated cirrhosis, bariatric surgery is contraindicated and needs to be discussed in the context of considering liver transplantation (**LoE 4, open recommendation, strong consensus**).

**Statement**

- Weight loss and optimised treatment of comorbidities before transplantation may confer a benefit in terms of cardiovascular morbidity, as well as long-term survival and reduced recurrence of severe MASLD after liver transplantation (**LoE 3, strong consensus**).

*In adults who received liver transplantation due to MASLD-related end-stage liver disease, can non-pharmacologic or pharmacologic measures reduce the risk of MASLD recurrence and improve long-term outcomes compared with no intervention?*

**Statements**

- In adults transplanted for MASLD-related end-stage liver disease, there is a high risk of recurrence of MASLD after liver transplantation, especially in adults with several metabolic risk factors (**LoE 3, strong consensus**).
- Adults transplanted for MASLD-related end-stage liver disease are also at risk of cardiovascular events and kidney disease which can negatively impact long-term survival (**LoE 2, strong consensus**).
- No specific issues related to MASLD are known to alter choice of medication or target values; the risk of recurrence of severe, fibrotic steatohepatitis reinforces the need to obtain optimal control of cardiometabolic risk factors (**LoE 5, strong consensus**).
- The benefit of controlling weight and obesity-related comorbidities on recurrence of MASLD post-liver transplant and on progression to advanced fibrosis is expected but needs to be demonstrated in dedicated trials (**LoE 5, strong consensus**).

**Recommendations**

- In adults transplanted for MASLD-related end-stage liver disease, therapeutic interventions to control obesity and related cardiometabolic complications are recommended (**LoE 3, strong recommendation, strong consensus**).
- After liver transplantation, standard non-pharmacological dietary and lifestyle interventions should be universally implemented; pharmacological management of hypertension, type 2 diabetes and lipid disorders should be implemented according to general clinical guidelines (**LoE 3, strong recommendation, strong consensus**).
- GLP1 receptor agonists may be considered to control weight and obesity-related comorbidities, although specific trials in transplant recipients are needed (**LoE 5, weak recommendation, strong consensus**).

## Future directions

Despite the enormous advances in the field, many important areas on the management of MASLD require further evidence to refine our clinical practice. Some of these areas, where further research is pressingly needed, are listed in Box 1.

## Supplementary Information

Below is the link to the electronic supplementary material.

ESM Tables (PDF 220 KB)

## Abbreviations

ALD: Alcohol-related liver disease
ALT: Alanine aminotransferase
CPG: Clinical Practice Guidelines
CSPH: Clinically significant portal hypertension
EASD: European Association for the Study of Diabetes
EASL: European Association for the Study of the Liver
EASO: European Association for the Study of Obesity
ELF: Enhanced liver fibrosis
FIB-4: Fibrosis-4 index
GLP1RA: Glucagon-like peptide 1 receptor agonists
HCC: Hepatocellular carcinoma
LoE: Level of evidence
LSM: Liver stiffness measurement
MASH: Metabolic dysfunction-associated steatohepatitis
MASLD: Metabolic dysfunction-associated steatotic liver disease
MetALD: Metabolic dysfunction-associated steatotic liver disease with moderate (increased) alcohol intake
NAFLD: Non-alcoholic fatty liver disease
SGLT2: Sodium–glucose cotransporter-2
SLD: Steatotic liver disease
VCTE: Vibration-controlled transient elastography
