# Publisher Correction: EASL–EASD–EASO Clinical Practice Guidelines on the management of metabolic dysfunction-associated steatotic liver disease (MASLD): Executive Summary

**DOI:** 10.1007/s00125-024-06258-6

**Published:** 2024-10-01

**Authors:** European Association for the Study of the Liver, European Association for the Study of Diabetes, European Association for the Study of Obesity, Frank Tacke, Paul Horn, Vincent Wai-Sun Wong, Vlad Ratziu, Elisabetta Bugianesi, Sven Francque, Shira Zelber-Sagi, Luca Valenti, Michael Roden, Fritz Schick, Hannele Yki-Järvinen, Amalia Gastaldelli, Roberto Vettor, Gema Frühbeck, Dror Dicker

**Affiliations:** 1 The EASL Building – Home of Hepatology, 7 rue Daubin, CH 1203 Geneva, Switzerland; 2 European Association for the Study of Diabetes e.V., Rheindorfer Weg 3, 40591 Düsseldorf, Germany; 3 EASO Brussels Office, The Library, 10 Square Ambiorix, 1000 Brussels, Belgium

**Publisher Correction: Diabetologia**

10.1007/s00125-024-06196-3

This paper was erroneously published under a non-Open Access license. The article is forthwith distributed under the terms of Creative Commons Attribution 4.0 International License. To view a copy of this licence, visit http://creativecommons.org/licenses/by/4.0/.

The article also incorrectly included ‘Clinical Practice Guideline Panel’ in the author line. The original article has been corrected.

