# Supplementary material for: EASL–EASD–EASO Clinical Practice Guidelines on the management of metabolic dysfunction-associated steatotic liver disease (MASLD): Executive Summary
Source: Diabetologia. 2024 Jun 13;67(11):2375–92. doi: 10.1007/s00125-024-06196-3 (PMC11519095; doi:10.1007/s00125-024-06196-3)
Supplement: Supplementary file 1 — ESM Tables (PDF 220 KB) [file 125_2024_6196_MOESM1_ESM.pdf]

EASL–EASD–EASO Clinical Practice Guidelines on the management of metabolic dysfunction-associated steatotic liver disease (MASLD): Executive Summary

European Association for the Study of the Liver, European Association for the Study of Diabetes and European Association for the Study of Obesity

*Clinical Practice Guideline Panel: chair: Frank Tacke; Assistant to Chair: Paul Horn; EASL panel members: Vincent Wai-Sun Wong, Vlad Ratziu, Elisabetta Bugianesi, Sven Francque, Shira Zelber-Sagi; EASL Governing Board representative: Luca Valenti; EASD co-chair: Michael Roden; EASD panel members: Fritz Schick, Hannele Yki-Järvinen, Amalia Gastaldelli; EASO co-chair: Roberto Vettor; EASO panel members: Gema Frühbeck, Dror Dicker

Corresponding author: European Association for the Study of Diabetes (EASD), email: info@easd.org

Table of content:

ESM Table 1. Grades of recommendation 3

ESM Table 2. Level of Evidence based on the Oxford Centre for Evidence-based Medicine (adapted). 3

ESM Table 3. Cardiometabolic risk factors in the definition of MASLD (1). 3

ESM Table 4. SLD due to aetiologies other than MASLD, MetALD or ALD. 4

ESM Table 5. Tools to quantify alcohol consumption and identify alcohol use disorders ……………………………………………………………………………………………5

ESM Table 6. Factors associated with a higher risk of HCC occurrence in non-cirrhotic MASLD. 5

ESM Table 7. Proposed tools for HCC risk-stratification in MASLD-related cirrhosis. 6

ESM Table 8. Summary of protein, energy, and dietary pattern recommendations for adults with cirrhosis as indicated by medical associations' Practice Guidance/ Guidelines. In addition, individuals with cirrhosis must abstain from alcohol. 7

ESM Table 9. Screening and management for comorbidities in individuals with MASLD before liver transplantation. Modified from (2, 3). 8

References 9

ESM Table 1. Grades of recommendation.

Grade	Wording	Criteria
Strong	Must, shall, should, is recommended<br>Shall not, should not, is not recommended	Evidence, consistency of studies, risk-benefit ratio, individual preferences, ethical obligations, feasibility
Weak or open	Can, may, is suggested<br>May not, is not suggested	

ESM Table 2. Level of Evidence based on the Oxford Centre for Evidence-based Medicine (adapted).

Level	Criteria	Simple model for high, intermediate and low evidence
1	Systematic Reviews (SR) (with homogeneity) of randomised controlled trials (RCT)	Further research is unlikely to change our confidence in the estimate of benefit and risk.
2	Randomised controlled trials (RCT) or observational studies with dramatic effects;<br>Systematic Reviews (SR) of lower quality studies (i.e. non-randomised, retrospective)	
3	Non-randomised controlled cohort/follow-up study/control arm of randomised trial (systematic review is generally better than an individual study)	Further research (if performed) is likely to have an impact on our confidence in the estimate of benefit and risk and may change the estimate.
4	Case-series, case-control, or historically controlled studies (systematic review is generally better than an individual study)	
5	Expert opinion (mechanism-based reasoning)	Any estimate of effect is uncertain.

*Level may be graded down based on study quality, imprecision, indirectness (study does not match questions), because of inconsistency between studies, or because the absolute effect size is very small; Level may be graded up if there is a large or very large effect size.

ESM Table 3. Cardiometabolic risk factors in the definition of MASLD (1).

Metabolic risk factor	Adult criteria
Overweight or obesity	Body mass index<br>≥25 kg/m^2 (≥23 kg/m^2 in people of Asian ethnicity)
	Waist circumference<br>≥94 cm in men and ≥80 cm in women (Europeans)<br>≥90 cm in men and ≥80 cm in women (South Asians and Chinese)<br>≥85 cm in men and ≥90 cm in women (Japanese)
Dysglycaemia or type 2 diabetes	<u>Prediabetes</u>: HbA_{1c} 39-47 mmol/mol (5.7-6.4%) or fasting plasma glucose 5.6-6.9 mmol/L (100-125 mg/dl) or 2-h plasma glucose during OGTT 7.8-11 mmol/L (140-199 mg/dl) *or*<br><u>Type 2 diabetes</u>: HbA_{1c} ≥48 mmol/mol (≥6.5%) or fasting plasma glucose ≥7.0 mmol/L (≥126 mg/dl) or 2-h plasma glucose during OGTT ≥11.1 mmol/L (≥200 mg/dl) *or*<br><u>Treatment for type 2 diabetes</u>
Plasma triglycerides	≥1.7 mmol/L (≥150 mg/dl) *or*<br>lipid-lowering treatment
HDL-cholesterol	≤1.0 mmol/L (≤39 mg/dl) in men and ≤1.3 mmol/L (≤50 mg/dl) in women *or*<br>lipid-lowering treatment
Blood pressure	≥130/85 mmHg *or* treatment for hypertension

HbA_{1c}, glycated haemoglobin; HDL, high-density lipoprotein; OGTT, oral glucose tolerance test

ESM Table 4. SLD due to aetiologies other than MASLD, MetALD or ALD.

Condition	Clinical/lab/histological findings	Diagnostic criteria
Hepatitis C virus-associated steatotic liver (genotype 3)	Low triglycerides, HCV genotype 3	HCV antibody with reflex testing HCV RNA and HCV genotype
Drug-induced Liver Disease (DILI)	Mostly microvescicular SLD	Investigate for drug intake:<br>• Corticosteroids<br>• Tamoxifen<br>• Amiodarone<br>• Irinotecan<br>• Methotrexate<br>• Lomitapide<br>• Valproate<br>• 5-Fluorouracil<br><br>Liver biopsy for confirmation
Hypobetalipoproteinaemia	Low triglycerides and cholesterol, fat malabsorption, vitamin A deficiency	ApoB level, genetic testing (*APOB, MTTP, PCSK-9*, targeted panel sequencing)
Lipodystrophy	Accumulation of fat in the visceral area and in the muscle (generically inherited or induced by HAART therapy)	CT scan or MRI, targeted panel sequencing for congenital lipodystrophies, MRI
LAL deficiency (Wolman disease, cholesteryl ester storage disease-CESD)	Elevated LDL-C and triglycerides, low HDL-C, hypersplenism, advanced fibrosis in young age, predominately microvesicular steatosis	Enzyme assay, genetic testing (LIPA)
Pregnancy associated	HELLP syndrome<br>Acute onset	Elevated liver enzymes and low platelets, haemolysis,<br>SLD at abdominal ultrasound
Wilson disease	Younger age, neuropsychiatric symptoms, low ceruloplasmin	24-h urine copper excretion; quantitative copper on liver biopsy, genetic testing (*ATP7B*)
Nutrient deficiency / malnutrition	Parenteral nutrition, bypass surgeries, bariatric surgery, anorexia	Nutrient levels
Celiac disease	Diarrhoea, iron deficiency, vitamins deficiency	Tissue transglutaminase IgA, duodenal biopsy
Endocrine diseases	Hypothyroidism, PCOS, growth hormone (GH) deficiency, panhypopituitarism (primary or secondary)	TSH, fT4, fT3, endocrine testing
Other inherited metabolic conditions	Early age and severe onset, absence of triggering factors, systemic involvement, positive history of advanced disease in first degree relatives	Targeted panel sequencing, whole exome sequencing (WES)

ALD, alcohol-related liver disease; ApoB, apolipoprotein B; fT3, free triiodothyronine; fT4, free thyroxine; HCV, hepatitis C virus; HAART, highly active antiretroviral therapy; HELLP, haemolysis, elevated liver enzymes and low platelets; LAL, lysosomal acid lipase; MASLD, metabolic dysfunction-associated steatotic liver disease; PCOS, polycystic ovary syndrome; SLD, steatotic liver disease; TSH, thyroid-stimulating hormone.

ESM Table 5. Tools to quantify alcohol consumption and identify alcohol use disorders.

Psychometric instruments	Biomarkers
• Medical history (including current and prior alcohol intake and drinking pattern)<br>• Quantity frequency questionnaires and diaries (Timeline Followback)<br>• Apps (*e.g.* Drinkaware)<br>• AUDIT (Alcohol Use Disorders Inventory Test) questionnaire – 10 questions<br>• AUDIT-C (shortened version, 3 questions)<br>• SIAC (Systematic Inventory of Alcohol consumption, 3 questions)	Indirect alcohol markers:<br>• GGT, AST, AST>ALT, MCV, %CDT<br>Direct alcohol markers:<br>• Alcohol (EtOH) in breath and/or serum<br>• Ethyl glucuronide (in urine or hair)<br>• Phosphatidylethanol<br>• Less established: ethyl sulfate, fatty acid ethyl esters

AST/ALT, aspartate/alanine aminotransferase; CDT, carbohydrate-deficient transferrin; GGT, gamma-glutamyltransferase; MCV, mean corpuscular volume.

ESM Table 6. Factors associated with a higher risk of HCC occurrence in non-cirrhotic MASLD.

Factor(s)
Presence and duration of T2D, obesity or both
Older age
Concurrent alcohol intake and/or smoking
Individuals with FIB-4 >3.25
Individuals with LSM >10 kPa and increasing change in LSM over time

ESM Table 7. Proposed tools for HCC risk-stratification in MASLD-related cirrhosis.

NITs	Formula/model variables/data	Study cohorts	Performance
HCC risk score	age + sex + platelet count + albumin + AST/√ALT available at: www.hccrisk.com	7,068 individuals with MASLD-related cirrhosis (407 incident HCC)<br>Mean FU: 3.7 years	Derivation cohort, C-index = 0.749<br>Validation cohort, C-index = 0.718
aMAP	(0.06 × age (years) + 0.89 × sex (M = 1; F = 0) + 0.48 × [($\log_{10}$ bilirubin (μmol/L) × 0.66 + albumin (g/L) × −0.085] – 0.01 × platelets (×10^3/mm^3) + 7.4) / 14.77 × 100	Overall individuals, n = 17,374 NVH validation cohort, n = 720<br><br>Total: 1,389 individuals with MASLD<br>Median FU: 4.61 years<br>F3-F4, n = 243 (17.5%)	NHV cohort:<br>Overall, C-index = 0.85 Cirrhosis, C-index = 0.61<br><br>Overall, C-index = 0.887 F3-F4, C-index = 0.754
GALAD	-10.08 + 0.09 × age (years) + 1.67 × gender (M = 1, F = 0) + 2.34 × $\log_{10}$ AFP (ng/ml) + 0.04 × AFP-L3 (%) + 1.33 × $\log_{10}$ DCP (ng/ml)	389 individuals with MASH (28 incident HCC)<br>Median FU: 167 months Cirrhosis, n = 77 (19.6%)	Higher GALAD score in individuals who developed HCC vs. individuals HCC-free as early as 1.5 years before HCC diagnosis
HEDS study	Risk factor associated to HCC development in individuals with cirrhosis:<br>Male gender (OR = 2.47; 95% CI 1.54–4.07; *p* <0.001)<br>Years with cirrhosis (OR = 1.06; 95% CI 1.02–1.1; *p* = 0.004),<br>Family h/o of liver cancer (OR = 2.69; 95% CI 1.11– 5.86; *p* = 0.02)<br>Age (OR = 1.17; 95% CI 1.03–1.33; *p* = 0.02)<br>Obesity (OR = 1.7; 95% CI, 1.08–2.73; *p* = 0.02)<br>AST (OR = 1.54; 95% CI 0.97–2.42; *p* = 0.06)<br>AFP (OR = 1.32; 95% CI 0.97–1.77; *p* = 0.07)<br>Albumin (OR = 0.7; 95% CI 0.46–1.07; *p* = 0.10)	Total: 1,325 individuals with cirrhosis (95 incident HCC)<br>Median FU: 2.2 years<br>MASLD, n = 327 (24.9%); 19 incident HCC	Performance of the multivariate set of risk factors: C-index = 0.73
THRI	age + etiology + gender + platelets<br>Age: <45 years = 0 points; 45–60 years, 50 points; >60 years, 100 points<br>Etiology: autoimmune/HCV SVR, 0 points; other, 36 points; MASLD, 54 points; HCV/HBV, 97 points Gender: Female = 0 points; Male = 80 points<br>Platelets: >200 = 0 points; 140–200 = 20 points; 80–130 = 70 points; <80 = 89 points<br>Total: 0–366 points	Derivation cohort: 2,079 individuals with cirrhosis<br>MASLD, n = 111 (5.3%)<br><br>Total: 2,491 individuals with cirrhosis<br>MASLD, n = 1,182 (48%)	10-year HCC incidence: low-risk (<120) = 3%;<br>medium-risk (120–240) = 10%<br>high-risk (>240) = 32%<br><br>C-index = 0.69
LiverRisk score	Linear regression model using age (years), blood glucose, cholesterol, AST, ALT, GGT and platelets<br><br>Available at: https://www.liverriskscore.com	Derivation cohort: 14,726 participants without known liver disease from the general population undergoing transient elastography for assessment of liver fibrosis.<br><br>Two external validation cohorts: 4,370 and 3,999 individuals	8-year risk of HCC in the high- risk group = 4.4%<br><br>8-year risk of HCC development in the two lower risk groups ≤ 0.1%

AFP, alpha-fetoprotein; DCP, Des-γ-carboxy prothrombin; FU, follow-up; OR, odds ratio.

ESM Table 8. **Summary of protein, energy, and dietary pattern recommendations for adults with cirrhosis as indicated by medical associations' Practice Guidance/ Guidelines. In addition, individuals with cirrhosis must abstain from alcohol.**

**Society/ Association**	**EASL 2019**		**ESPEN 2019 and joint ESPEN/UEG guideline 2023**		**AASLD 2021**	
**BMI status†**	**Mixed BMIs**	**Obese BMI >30 kg/m²)**	**Mixed BMIs**	**Obese (BMI >30 kg/m²)**	**Non-obese**	**Obese (non-hospitalised, clinically stable)**
Daily energy	35 kcal/kg actual BW (in nonobese individuals)	>5–10% WR, moderately hypocaloric diet (-500–800 kcal/d)	30-35 kcal/kg only for DC. Regular energy requirements in CC	WR. No need for increased energy intake	≥35 kcal/kg body weight/day	25-35 kcal/kg/day for individuals with BMI 30-40 kg/m², and 20-25 kcal/kg/day for individuals with BMI ≥40 kg/m². WR if medically required, under the supervision of a multidisciplinary team. Caution applied to prescribing weight loss in decompensated cirrhosis.
Daily protein	1.2–1.5 g/kg actual BW	>1.5 g / kg IBW	1.2 g/kg (for non-malnourished individuals with CC) to 1.5 g/kg (to malnourished and/or sarcopenic cirrhosis)	Individuals with overweight or obesity and compensated cirrhosis: 1.2 g/kg ABW/d. Individuals with overweight or obesity and compensated cirrhosis undergoing weight-loss programs: 1.2–1.5 g/kg ABW/d. Individuals with overweight or obesity and compensated cirrhosis and malnutrition or sarcopenia: 1.5 g/kg ABW/d.	1.2-1.5 g/kg IBW. For individuals with cirrhosis who are critically ill, a target of 1.2-2.0 g/kg IBW	Intake of target protein (1.2-1.5 g/kg/day) and physical activity are required to reduce the loss of muscle contractile function and muscle mass that can occur with weight loss.
Meal patterns	Split food intake into 3 main meals and 3 snacks		Three to five meals a day and a late evening snack		Maximum interval of 3-4 hours between nutritional intake while awake. To minimise nocturnal fasting time, an early breakfast and/or late-evening snack recommended	
Dietary protein source in case of HE	Individuals may tolerate animal protein (meat) less well than vegetable protein (beans, peas etc.) and dairy proteins		In individuals who are protein "intolerant", vegetable proteins should be used		A diverse range of protein sources, including vegetable and dairy products, should be encouraged.	

ABW, adjusted body weight; BMI, body mass index; CC, compensated cirrhosis; DC, decompensated cirrhosis; HE, hepatic encephalopathy; IBW, ideal body weight; WR, weight reduction.

ABW = reference body weight (in which BMI = 25) + 0.33*(actual body weight - reference body weight).

†In a case of fluid retention, body weight should be corrected by evaluating the individual's dry weight.

ESM Table 9. Screening and management for comorbidities in individuals with MASLD before liver transplantation. Modified from (2, 3).

Condition	Recommendation
Type 2 diabetes	• Screen for impaired fasting glucose (IFG) or glucose tolerance (IGT) and/or T2D (OGTT, HbA_{1c})<br>• Achieve good glycaemic control before LT<br>• Preferentially use weight-lowering (*e.g.* SGLT2 inhibitors, GLP1RA) or weight-neutral (*e.g.* metformin) glucose lowering medication, considering risk of other diabetes complications, if liver and/or renal function allow this
Nutrition	• Assess nutritional status before LT<br>• Assess alcohol consumption<br>• Healthy diet, physical exercise and lifestyle modification (including weight reduction in individuals with obesity) represent pillars in pre-LT management
Cardiovascular	• Pre-LT cardiovascular risk stratification is mandatory<br>• Risk-adapted algorithm of cardiac work-up should be followed (see Fig. 5)<br>• LT candidates with cardiovascular risk should be managed with goal-directed medical management (*e.g.*, statins, anti-platelet agents, beta blockers, RAAS blockers), based on the stage of cirrhosis and renal function
Kidney	• Kidney function should be adequately monitored before LT<br>• Comedications need to be adjusted (or replaced) dependent on kidney function
Malignancies	• Screening for pre-LT malignancies should follow the same protocols applied to individuals with non-MASLD related cirrhosis (including gastrointestinal and genital cancers)

GLP1RA, glucagon-like peptide-1 receptor agonist; HbA_{1c}, glycated haemoglobin; LT, liver transplantation; MASLD, metabolic dysfunction-associated steatotic liver disease; OGTT, oral glucose tolerance test; RAAS, renin-angiotensin-aldosterone system; SGLT2, sodium-glucose cotransporter-2.

References

1. Rinella ME, Lazarus JV, Ratziu V, Francque SM, Sanyal AJ, Kanwal F, et al. A multi-society Delphi consensus statement on new fatty liver disease nomenclature. J Hepatol. 2023;79(6):1542-56.

2. Tsochatzis EA, Watt KD, VanWagner LB, Verna EC, Berzigotti A. Evaluation of recipients with significant comorbidity - Patients with cardiovascular disease. J Hepatol. 2023;78(6):1089-104.

3. Burra P, Becchetti C, Germani G. NAFLD and liver transplantation: Disease burden, current management and future challenges. JHEP Rep. 2020;2(6):100192.
